# Identification of Historical Veziragasi Aqueduct Using the Operational Modal Analysis

**DOI:** 10.1155/2014/518608

**Published:** 2014-01-08

**Authors:** E. Ercan, A. Nuhoglu

**Affiliations:** Department of Civil Engineering, Ege University, 35100 İzmir, Turkey

## Abstract

This paper describes the results of a model updating study conducted on a historical aqueduct, called Veziragasi, in Turkey. The output-only modal identification results obtained from ambient vibration measurements of the structure were used to update a finite element model of the structure. For the purposes of developing a solid model of the structure, the dimensions of the structure, defects, and material degradations in the structure were determined in detail by making a measurement survey. For evaluation of the material properties of the structure, nondestructive and destructive testing methods were applied. The modal analysis of the structure was calculated by FEM. Then, a nondestructive dynamic test as well as operational modal analysis was carried out and dynamic properties were extracted. The natural frequencies and corresponding mode shapes were determined from both theoretical and experimental modal analyses and compared with each other. A good harmony was attained between mode shapes, but there were some differences between natural frequencies. The sources of the differences were introduced and the FEM model was updated by changing material parameters and boundary conditions. Finally, the real analytical model of the aqueduct was put forward and the results were discussed.

## 1. Introduction

The variety and natural availability of the materials needed for masonry and the choice of laying pieces of stones or bricks on top of each other with or without mortar have made this technique easy and common. Additionally, masonry has important characteristics such as aesthetics, solidity, durability, easy maintenance, versatility, and fire protection as discussed by Lourenço [1]. However the lack of insight and models for the complex behavior of units, mortar, joints, and masonry as a composite material has made analysis of masonry difficult. Existing calculation methods are mainly of an empirical and traditional nature and the use of numerical tools for the analysis or design of masonry structures is rather incipient as discussed by Lourenço [2]. Historical masonry structures have been particularly vulnerable to earthquakes and winds for thousands of years. The preservation of historical structures is considered to be a fundamental issue in the cultural life of modern societies as a consequence of the failure of preventing some of them from collapsing in past decades [3]. For this reason, the conservation and the structural safety assessment of historical structures have become an increasing concern. There are many studies concerning this topic in the literature including both analytical and experimental investigations of these structures.

Bayraktar et al. [4] described the results of an ambient vibration test and operational modal analysis carried out on the historical masonry bell-tower of the Hagia Sophia church in Trabzon, Turkey. Bernardeschi et al. [5] described the numerical techniques implemented in the finite element code for structural analysis of Buti's masonry bell-tower, a medieval structure located on the Pisa mountains. Two different load conditions were taken into consideration in the numerical analysis of the tower: the first was its own weight, and the second was an earthquake load in addition to its own weight. Bayraktar et al. [6] investigated the nonlinear seismic performance of the Mikron arch bridge, a nineteenth century Ottoman-era structure built over the Firtina river near Rize, Turkey, using ambient vibrations. Firstly, a finite element analysis of the Mikron arch bridge was conducted, then the bridge was subjected to ambient vibration testing, and the vibratory response was obtained. The investigators then used enhanced frequency domain decomposition and stochastic subspace identification techniques to extract the experimental natural frequencies, mode shapes, and damping ratios from these measurements. The experimental results were compared with those obtained by the linear finite element analysis of the bridge. A good agreement between the mode shapes was observed during this comparison, though this was not the case for natural frequencies. The boundary conditions and material properties of the linear finite element model of the Mikron arch bridge were readjusted using the vibration test results and the analytical model was updated.

It is seen that the ambient vibration test has been preferred in many attempts to determine the dynamic characteristics of structures. The method is especially preferred for testing of historical structures because no excitation equipment is needed. As environmental excitations such as wind, traffic, and human influences are always present, the test implies a minimum interference with the normal use of the structures. Furthermore, the ambient vibration test does not have any negative effect on the structures.

**Figure 1:**
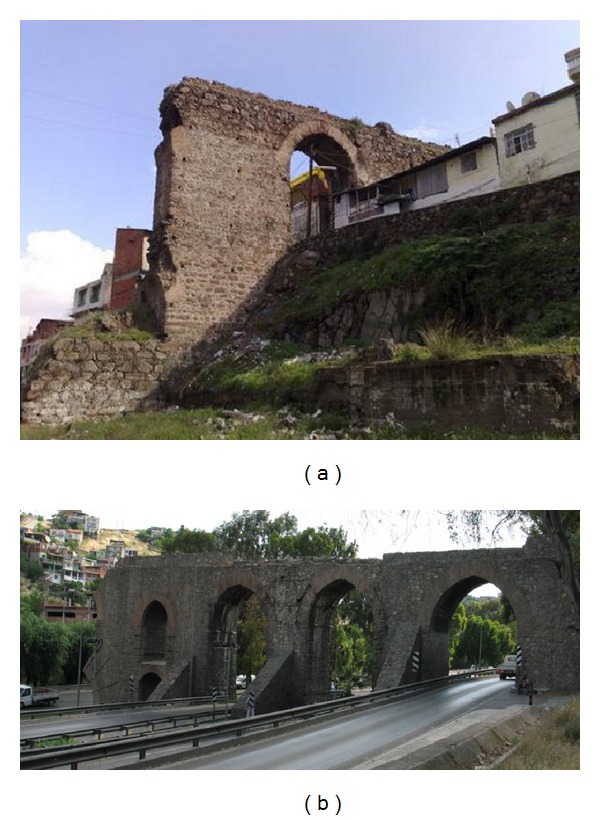
Vezirağasi aqueduct.

In this study, an Ottoman aqueduct, Veziragasi, (Figure 1) was investigated. An aqueduct is a water supply or navigable channel constructed to convey water. Although particularly associated with the Romans, aqueducts were devised much earlier in Greece, in the Near East, and on the Indian subcontinent, where peoples such as the Egyptians and Harappans built sophisticated irrigation systems. Like most other structures, aqueducts were built using the masonry technique.

The Ottoman “Vezir System,” conveying the waters of springs near Buca, Southeast of Izmir, and crossing Melez creek via a high aqueduct is called Veziragası or Vezir. The Vezir aqueduct was constructed in 1678 by the order of Grand Vizier Köprülü Fazıl Ahmet Paşa. The stone used in the construction of the structure is roughly cut andesite, except for the arches, which are made from clay brick with a thick mortar. In the study, firstly, the dimensions of the structure, defects such as cracks and material degradations on the structure, and the materials used in different parts were determined in detail by making a measurement survey. Secondly, the material properties of the components of the masonry were determined for the finite element method (FEM) using uniaxial compressive and indirect tension tests. Nondestructive testing (NDT) methods such as impact-echo, ultrasonic-echo, and ultrasonic pulse velocity testing methods were also used for evaluation of material properties. The SIBIE (stack imaging of spectral amplitudes based on impact-echo) technique was applied to evaluate the inner structure of the aqueduct. The solid model of the structure was then developed and the modal analysis of the structure was calculated by FEM. A nondestructive dynamic test and operational modal analysis (OMA) were then administered and the dynamic properties of the aqueduct were extracted. The results from OMA and the results from FEM were compared and according to the feedback, the FEM model was updated by changing the material parameters and boundary conditions. Finally, the real material properties were determined.

## 2. Descriptions of the Structure, History Investigation, and Measurement Survey

The Vezir aqueduct system brings water to the fountains of the city of İzmir. The Vezir aqueduct was constructed in 1678 by the order of Grand Vizier Köprülü Fazıl Ahmet Paşa. Grand Vizier Köprülü Fazıl Ahmet Paşa funded the construction of this water system by himself so the system was called Vezir Water System, as discussed by Aktepe [7]. Today Vezir aqueduct is located from North to East along Yeşildere Street and on the hillside of the Kadifekale. The walls of the structure are stone masonry with grey and pink andesite and mortar joints. The span arches are brick masonry with thick mortar. The three arches of the North section have extensive cracks.

The aqueduct was previously 150 meters in length but 45 meters of the Vezir aqueduct collapsed and today 85 meters with 4 spans at one side of the North section and a 20-meter section with one span at the other side of the South section remain. The North section has a length of 85 meters, a width of 3.5 meters, and a height of 8 meters. The span lengths from North to South are 5.20 m, 5.33 m, 6.00 m, and 3.5 m, respectively.

### 2.1. Material Tests on Stone, Brick, and Mortar

**Figure 2:**
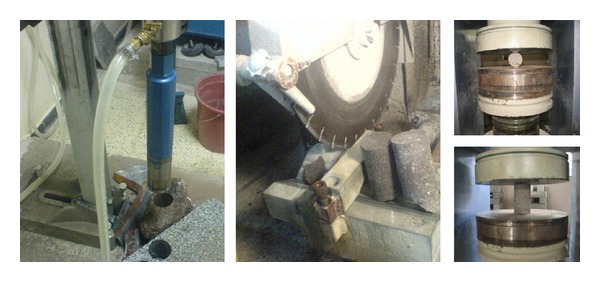
Drilling out core samples, cutting edges, uniaxial compression, and Brazilian test.

In order to determine the parameters needed for finite element modeling, material nondestructive and destructive tests were administered on the constituents of the masonry. Stones and bricks from the structure were taken and destructive tests were carried out. Samples strong enough from which to cut out core samples of diameter 54 mm for stone and diameter 25 mm for bricks were selected. Indirect tension tests (Brazilian test) and uniaxial compression (UAC) tests were carried out as discussed in TS 699 and Ulusay et al. [8, 9] (Figure 2). The average results are shown in Table 1.

**Figure 3:**
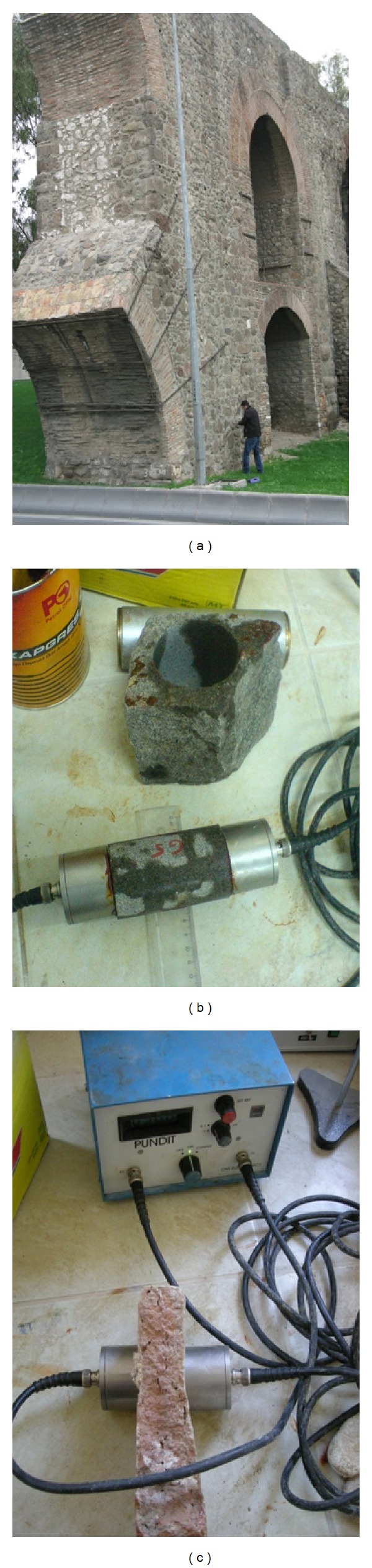
*In situ* Schmidt test and ultrasonic wave velocity test on stone and brick.

For the nondestructive tests, before taking the samples to the laboratory, L and LB type Proceq Schmidt hammers were used to find the surface hardness values (rebound value) of the stone and clay brick samples, respectively. The Schmidt hammer test was also applied to the stones and clay bricks of the whole structure (Figure 3). The compressive strength was calculated from the “rebound (R) value-compressive strength scheme” of Ulusay et al. [9]. In the laboratory, before indirect and uniaxial compression tests were applied to the stone and clay brick samples, ultrasonic wave velocity tests had been conducted using pundit type equipment (Figure 3). The modulus of elasticity values of the stone samples was also determined by (1) given in ASTM [10]:

(1) $$\begin{array}{c} E=V^{2}\rho \left(1+m\right)\times \frac{\left(1-2m\right)}{\left(1-m\right)}, \end{array}$$

where *V*, *ρ*, and *m* are ultrasonic pulse velocity, density, and Poisson's ratio, respectively. The Poisson's ratio is taken to be 0.21 for the andesite stone. The estimated modulus of elasticity values from ultravelocity tests is higher than the values obtained through destructive tests (Table 1).

The mortar samples were weak and too small for drilling so only point load tests could be applied on arbitrary-shaped samples. By use of the point load index, the uniaxial compressive strengths of mortars were estimated. The average estimated uniaxial strength of mortars was calculated to be 6.25 MPa from point load tests. Tensile strength and modulus of elasticity of mortar were also estimated from the literature with the aid of point load test results. The tensile strength and modulus of elasticity *σ*_*t*_, 0.79 MPa, and *E*, 150 MPa, were taken, respectively. The density of the mortar was calculated to be *ρ* = 1.70 gr/cm^3^.

## 3. Determination of Material Parameters for Analytical Model

Masonry is a composite and this composite material consists of two or more different constituent materials. By the use of the homogenization approach, the behaviors of mortar and stone/brick were assumed to act together so the overall behavior of the composite media has been taken into account. While determining the elastic parameters of the masonry aqueduct, the homogenization equations which depend on the strength parameters of constituents were used. The aqueduct has two types of masonry: stone masonry MS and brick masonry BS.

### 3.1. Determination of Material Parameters of MS and BS from Destructive Test Results

The compressive strength of masonry is determined by (2) as described by Eurocode 6 [11]:

(2) $$\begin{array}{c} f_{k}=K\cdot f_{b}^{0.65}\cdot f_{m}^{0.25}, \end{array}$$

where *K* is a constant, *f*_*b*_ is the compressive strength of stone or brick, and *f*_*m*_ is compressive strength of mortar. *K* is in the range from 0.4 to 0.6 and depends on the morphology of the masonry as described by Eurocode 6 [11]. *K* was taken to be 0.5 in this study.

The modulus of elasticity of masonry was determined by the use of (3) as described by Lourenço [2]:

(3) $$\begin{array}{c} E=\frac{t_{m}+t_{m}}{\left(t_{m}/E_{m}\right)+\left(t_{u}/E_{u}\right)}\rho , \end{array}$$

where *t*_*m*_,  *t*_*u*_,  *E*_*m*_,  and *E*_*u*_ are the thickness of mortar and height of the unit (stone or clay brick); the coefficient *ρ* varies with the bond between mortar and unit and was taken to be 0.5 for this study as described by Lourenço [2].

The shear modulus can be taken to be 40% of the modulus of elasticity as described by Eurocode 6 [11]. The tensile strength of masonry can be taken to be 10% of compressive strength as described by Koçak [12].

The densities of BM and SM were calculated to be 2.1 kg/cm^3^ and 1.75 kg/cm^3^, respectively. The Poisson ratio was taken to be 0.17 for masonry as described by Koçak [12]. The elastic material parameters of SM and BM for the finite element model are shown in Table 2.

### 3.2. Impact-Echo Tests

Impact-echo is one of the nondestructive testing methods for concrete based on multiple reflections of an acoustical wave between the test surface of concrete and an interface between materials with different mechanical impedances. An impact load is applied at the surface of the concrete and the vibrations caused by this impact are recorded by a receiver. As a result, a waveform is built up in the time domain. In the traditional impact-echo analysis, this waveform is transformed into the frequency domain by applying FFT. Peak frequencies are identified in the frequency spectrum and corresponding depth is calculated by the given formula in (4), where *C*_*p*_ is longitudinal wave velocity, *f* is the measured frequency, and *d* is the corresponding depth as described by Sansolone [13] and Ata et al. [14]:

(4) d=Cp2f.

However, in most of the practical applications, due to the complex information existing in the data, it is difficult to interpret the frequency spectrum. Consequently, the SIBIE (stack imaging of spectral amplitudes based on impact-echo) procedure has been developed to improve the impact-echo method. The impact-echo method has been utilised for the evaluation of masonry structures. In this study, a stone masonry bridge was tested by carrying out the impact-echo and SIBIE procedures.

#### 3.2.1. SIBIE Procedure

Based on the inverse scattering theory in elastodynamics as described by Nakahara and Kitahara [15], the SIBIE procedure was developed at Kumamoto University by Ohtsu and Watanabe [16] and Ata et al. [14]. This is an imaging technique for detecting waveforms in the frequency domain. In this study, the theory of SIBIE will not be mentioned.

#### 3.2.2. Impact-Echo Tests Results

**Figure 4:**
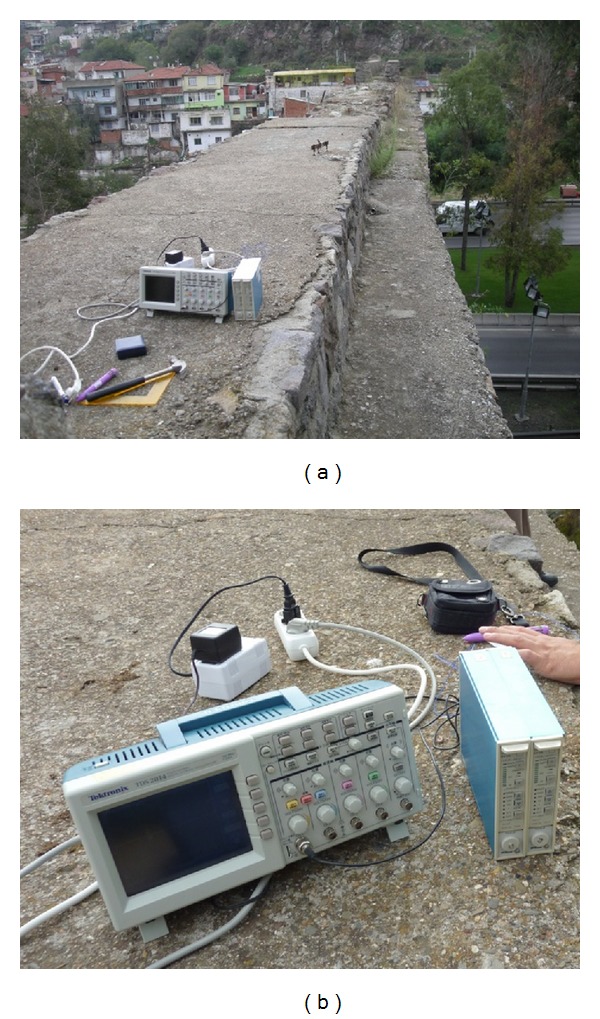
Impact-echo test on aqueduct and digital storage oscilloscope.

The clay masonry arch tested is shown in Figure 4. The tests were carried out at several locations on the arches and walls of aqueduct. A hammer with spherical hitting edge was used in the tests to generate elastic waves. Since the depth of the section to be tested was large, a hammer was used instead of a steel sphere to generate a high impact energy. A digital storage oscilloscope (Tektronix TDS 2014) and an accelerometer (Figure 4) were used to detect surface displacements caused by reflections of the elastic waves. The frequency range of the accelerometer system was from DC to 50 kHz. The Fourier spectra of accelerations were analyzed by FFT (Fast Fourier Transform). Sampling time was 4 *μ*sec and the number of digitized data for each waveform was 2048. The cross-section of the bridge part tested was divided into square elements to perform SIBIE analysis. In this study, the size of square mesh for SIBIE analysis was set to 10 mm.

**Figure 5:**
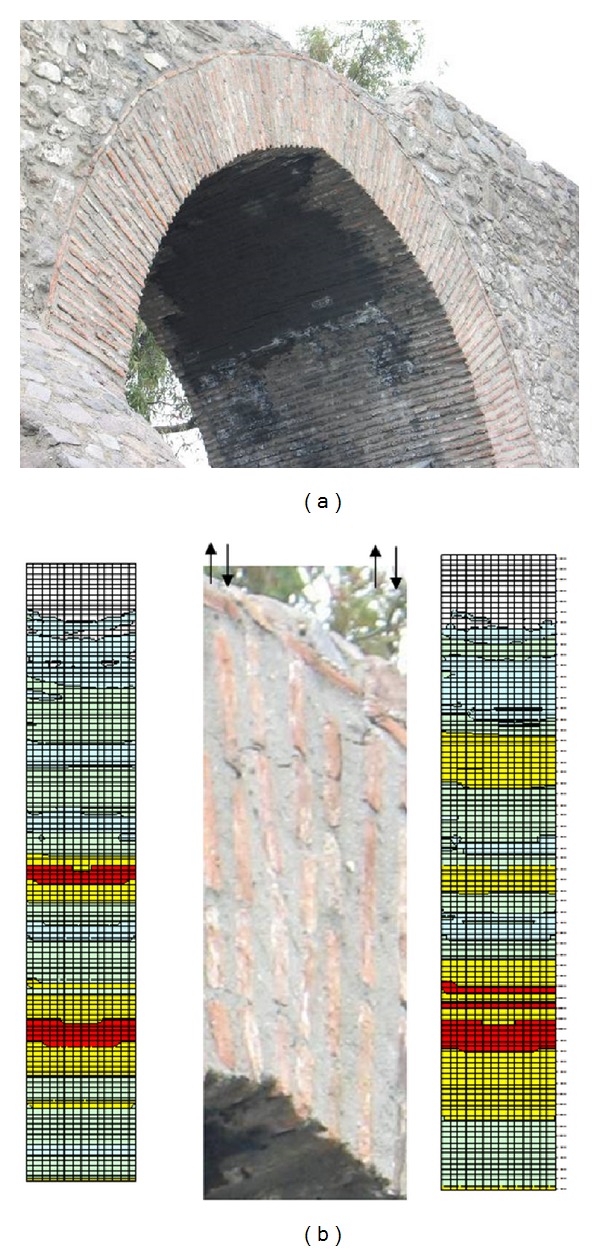
Two impact-echo test results on arc.

The results of the SIBIE analysis of the impact applied above the center of the arch are depicted in Figure 5. The SIBIE figure corresponds to the cross-section of the element that is tested. Here, the red color zones indicate the higher reflection due to the interface between materials with different mechanical impedances. In the figure, impact and detection locations are indicated with arrows. The distance between impact and detection was selected to be 5 cm. The depth of the section tested was 110 cm. The back wall reflection of the yellow color zone cannot be clearly observed in the SIBIE figure. The red color regions are clearly observed corresponding to the material interfaces. These reflections are due to extensive cracks in the arch which must be considered in the analytical model of the aqueduct.

According to the test results, the discontinuities in the structure appeared in the SIBIE results as relevant regions (red zones in this case). By evaluating the results, it can be said that there are some flaws between layers. Some flaws could be seen by side but SIBIE results show that there were others inside the structure as well. The size of these flaws was estimated to be about 3-4 cm and occurred because of delamination of unit and mortar. These flaws decrease the strength of the material and thus affect the durability of the structure and these factors are considered in the FEM model of the structure.

## 4. Eigen Values and Mode Shapes of Vezir Aqueduct

**Figure 6:**
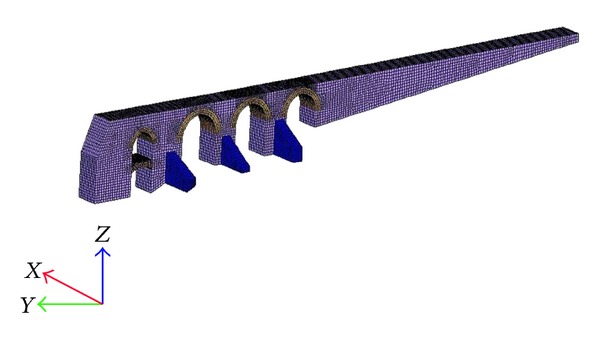
3D FE model of Vezir aqueduct.

For the Eigen values and mode shapes of the structure, a commercial finite element program SAP 2000 [17] was used. First of all, with the help of the measurement survey data, the 3D geometry of the Veziragasi aqueduct was defined by 37376 solid elements (Figure 6) and the calculated material parameters of SM (stone masonry) and BM (brick masonry) were defined to the program and the FE model was created. After the analysis was completed, the theoretical modal shapes of the structure were gathered. The results are shown in Table 3.

## 5. Operational Modal Analysis of Vezir Aqueduct 

The dynamic behavior of structures is related to their modal characteristics such as natural frequencies, mode shapes, and damping ratios. This dynamic behavior is a combination of many factors including assumptions in the design criteria and construction and uncertainties in the geometrical and material properties. Modal testing is a method to estimate the natural frequencies and mode shapes. Various methods, including both time and frequency domain-based procedures, are available for extracting modal information from the dynamic response of a structure and the corresponding input excitation. The process of establishing the dynamic characteristics of a system from an experimental model is called “system identification.” In this study operational modal analysis (OMA) is used for system identification. In OMA, accelerometers are placed on the structure and the structure is excited by an unknown input force such as traffic, wind, and seismic loads caused by ambience and the responses of the structure are measured by these accelerometers.

The operational modal analysis ambient excitation does not lend itself to frequency response functions (FRFs) or impulse response function calculations because the input force is not measured. Therefore, a modal identification procedure will need to base itself on output-only data. Some modal parameter identification methods have been developed. In this study, the peak picking (PP) method from the power spectral densities (PSDs) and SSI methods were used for OMAs. The physics background of all these methods is quite similar, but a few implementation aspects such as data reduction, type of equation solvers, and sequence of matrix operations are different. In this study, the theory of OMA will not be mentioned.

**Figure 7:**
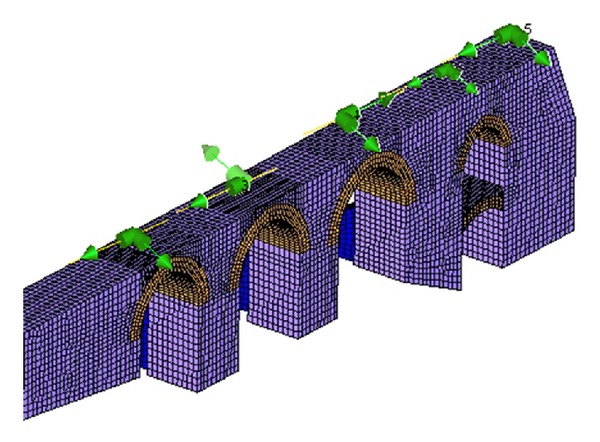
Uniaxial accelerometers placed to the top of the aqueduct alongside.

**Figure 8:**
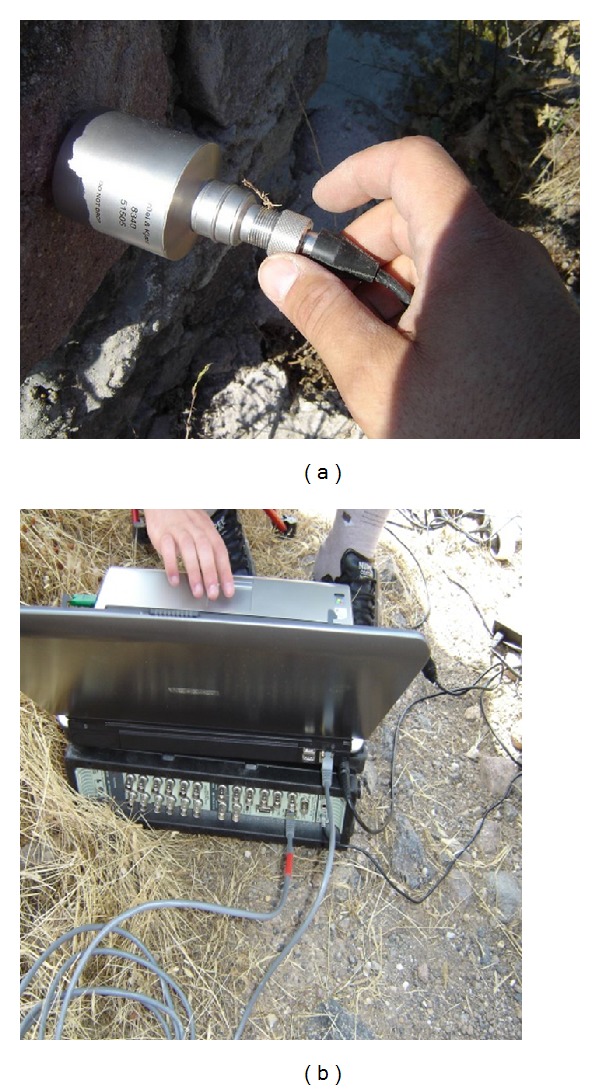
Uniaxial accelerometers and 17-channel data acquisition system.

**Figure 9:**
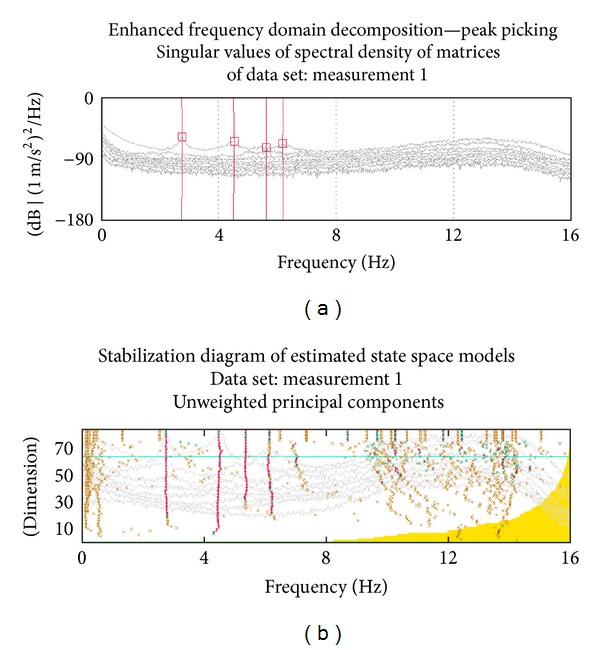
The frequency response function (PP method) and stabilization diagram of the measurement (SSI method).

Two ambient vibration tests were conducted for reliability on the aqueduct to determine its dynamic characteristics. 10 uniaxial accelerometers were used in OMA and these were placed on the top of the aqueduct alongside and the model was prepared for OMA (Figure 7). Signals acquired from accelerometers were gathered in the 17-channel data acquisition system (Brüel and Kjaer 3560) and were sent to Pulse software for further processing (Figure 8). Modal parameters were then extracted using OMA software [18]. The ambient vibration tests were conducted under environmental loads such as wind effects and traffic. The OMA, PP, and SSI methods (Figure 9) were used to determine the natural frequencies, mode shapes, and damping ratios of the aqueduct. The results from test 1 and test 2 are shown in Table 3. Frequencies obtained with the SSI method are nearly the same with those found in the PP method but a little bit different from the FEM results. Differences between the analytical and experimental results come from some uncertain parameters of the masonry.

## 6. Evaluation of Analytical and Experimental Results 

The modal analyses results indicated that the initial theoretical frequencies were greater than the experimental frequencies, while the mode shapes had a good harmony. The possible reasons for this are the unknown mechanical properties of the masonry, cracks, and boundary conditions of the base. By changing some boundaries and the elasticity modulus of the foundation of the aqueduct, the FEM model was updated and frequency values from FEM had become nearly the same as the experimental values as shown in Table 4.

The study also clearly showed that the ambient vibration test is a very effective method for determining the dynamic characteristics of historical structures under operational conditions. In this study, the natural frequencies, mode shapes, and modal damping ratios of the Vezir aqueduct were obtained using the ambient vibrations produced by traffic and wind. This was a nondestructive test for the aqueduct. Structural health can be assessed depending on the changes in dynamic characteristics of the aqueduct. If the natural frequencies and mode shapes of the aqueduct are measured again later, structural health can be evaluated by considering the changes.

## 7. Conclusions

The conventional testing methods and empirical formulas given in codes and standards for determining the material parameters of the masonry structures can be considered sufficient. On the other hand, by using the OMA results, the material properties and boundary conditions assigned in the FE model can be modified to have real structural behavior. At first, the modal shapes extracted from FEA and OMA were in good agreement with each other but there was a little bit of difference in the frequency values. By changing the boundary conditions and modulus of elasticity using the OMA results, this difference was reduced and the 3D FE model of the structure was modified. With the help of the updated FE model, the structure can be tested for different earthquakes which have happened in the past and the structural health of the aqueduct can be understood.

## Figures and Tables

**Table 1:** Test results of stone and clay brick.

Sample	Number of samples	Density *ρ* (g/cm^3^)	Rebound value, *R*	Compressive strength from *R* (Mpa)	Compressive strength from UAC (Mpa)	*E* from UAC (Gpa)	Tensile strength from Brazilian test (Mpa)	Ultravel. (m/sec)	*E* from U. V. (Gpa)
Stone (standard deviation)	25	2.43 (0.8)	53.49 (2.7)	82	53.35 (3.4)	11.59 (0.4)	6.09 (1.81)	4030 (220)	16.24
Brick (standard deviation)	11	1.79 (0.1)	30.74 (4.4)	24	10.41 (2.1)	0.81 (0.06)	1.43 (0.08)	2739 (180)	—

**Table 2:** Material parameters of masonry for FE model.

Material parameter	Stone masonry	Brick masonry
Compressive strength (MPa)	10.49	3.62
Tensile strength (MPa)	1.05	0.36
Modulus of elasticity (MPa)	871	201
Shear modulus	326	80.4
Density (kg/m^3^)	2100	1750
Poisson ratio	0.17	0.17

**Table 3:** Comparison of analytical and experimental modal parameters.

Frequency number	Analytical modal parameters (Hz)	PP method (Hz)		SSI method (Hz)		Damping ratios (%)	
		Test 1	Test 2	Test 1	Test 2	Test 1	Test 2
1	2.877	2.769	2.778	2.758	2.758	1.896	1.896
2	4.205	4.513	4.542	4.556	4.52	3.304	3.304
3	5.555	5.413	5.405	5.39	5.375	3.846	3.846
4	5.83	6.198	6.149	6.08	6.099	2.723	2.723
5	7.3699	Not extracted	7.035	Not extracted	7.015	Not extracted	2.105

**Table 4:** Updated and experimental modal frequencies.

Frequency	Analytical (SAP 2000)	Updated frequencies (SAP 2000)	Experimental frequencies (PP)	Experimental frequencies (SSI)
1	2.877	2.755	2.758	2.759
2	4.2050	4.521	4.556	4.515
3	5.555	5.410	5.391	5.398
4	5.831	5.971	6.089	6.219
